# Applying Human Factors Principles to Mitigate Usability Issues Related to Embedded Assumptions in Health Information Technology Design

**DOI:** 10.2196/humanfactors.3524

**Published:** 2014-12-18

**Authors:** Michael C Gibbons, Svetlana Z Lowry, Emily S Patterson

**Affiliations:** ^1^ Johns Hopkins University Departments of Medicine, Public Health, and Health Informatics Baltimore, MD United States; ^2^ Johns Hopkins Urban Health Institute Baltimore, MD United States; ^3^ National Institute of Standards and Technology Information Access Division, Information Technology Laboratory Gaithersburg, MD United States; ^4^ The Ohio State University College of Medicine Division of Health Information Management and Systems School of Health and Rehabilitation Sciences Columbus, OH United States

**Keywords:** cultural ergonomics, culturally informed design, EHR, health care disparities, health information technology, human factors, patient portal, patient safety, usability, workflow

## Abstract

**Background:**

There is growing recognition that design flaws in health information technology (HIT) lead to increased cognitive work, impact workflows, and produce other undesirable user experiences that contribute to usability issues and, in some cases, patient harm. These usability issues may in turn contribute to HIT utilization disparities and patient safety concerns, particularly among “non-typical” HIT users and their health care providers. Health care disparities are associated with poor health outcomes, premature death, and increased health care costs. HIT has the potential to reduce these disparate outcomes. In the computer science field, it has long been recognized that embedded cultural assumptions can reduce the usability, usefulness, and safety of HIT systems for populations whose characteristics differ from “stereotypical” users. Among these non-typical users, inappropriate embedded design assumptions may contribute to health care disparities. It is unclear how to address potentially inappropriate embedded HIT design assumptions once detected.

**Objective:**

The objective of this paper is to explain HIT universal design principles derived from the human factors engineering literature that can help to overcome potential usability and/or patient safety issues that are associated with unrecognized, embedded assumptions about cultural groups when designing HIT systems.

**Methods:**

Existing best practices, guidance, and standards in software usability and accessibility were subjected to a 5-step expert review process to identify and summarize those best practices, guidance, and standards that could help identify and/or address embedded design assumptions in HIT that could negatively impact patient safety, particularly for non-majority HIT user populations. An iterative consensus-based process was then used to derive evidence-based design principles from the data to address potentially inappropriate embedded cultural assumptions.

**Results:**

Design principles that may help identify and address embedded HIT design assumptions are available in the existing literature.

**Conclusions:**

Evidence-based HIT design principles derived from existing human factors and informatics literature can help HIT developers identify and address embedded cultural assumptions that may underlie HIT-associated usability and patient safety concerns as well as health care disparities.

## Introduction

Pervasive and intractable health care disparities have been convincingly documented at all levels of the US health care system. Health care disparities are associated with poor health outcomes, premature death, and increased health care costs [1,2]. Although a large body of work has demonstrated the existence of these disparities, there has not been any significant systematic and sustained improvement over time [3]. Furthermore, several national trends in the United States suggest that the scope and magnitude of these disparities are likely to increase, including the growth of racial, ethnic minority, immigrant, senior populations (eg, aging baby boomers), an aging health care workforce, and a significant problem with health literacy and English-language fluency among US residents [4]. These disparities are associated with excess morbidity and mortality, as well as increased health care costs and patient harm, among affected populations and society in general. It has been estimated that the combined direct and indirect cost of health disparities in the United States was $1.24 trillion between 2003 and 2006 [2]. Despite many efforts to date, there has been no systematic and sustained reduction in any health disparity at the national level [5].

A number of federal agencies have called for an increasing role for health information technology (HIT) in health care delivery as a way to address health care disparities, in addition to improving efficiency, quality, and patient safety [6]. The Center for Medicaid & Medicare Services “meaningful use” program promotes the use of electronic health records (EHRs), primarily through financial incentives. Meaningful use has been implemented in three stages. Stages 1 and 2 are focused on introducing EHR use and integration within a health information exchange system [7]. To the extent that EHRs are increasingly becoming decision-making support tools for both providers and patients through associated patient portals, it will be important to both understand the impact of and effectively address utilization differences associated with embedded cultural assumptions. Indeed, reported disparities in EHR patient portal use may be associated with such inappropriate cultural assumptions [8-11].

In the United States, populations affected by disparities include racial and ethnic minorities; persons of low socioeconomic status; and those who have limited English proficiency, are living with disabilities, and are over the age of 65 [12]. The culturally informed design framework [13] is intended to provide conceptual guidance for designers of HIT who are interested in considering cultural factors in their system designs. The authors of this framework articulated 4 design dimensions in which cultural factors could impact usability: (1) technology platform, (2) technology functionality, (3) information content, and (4) HIT system-user interface. For example, regarding the technology platform, African Americans and Latinos are much more likely than other subpopulations to use mobile devices as their primary means of accessing the Web [14] when they use any technology. In terms of functionality, low-income racial and ethnic minorities are more likely than other populations not to use any technology at all. Among these individuals, 32% cite usability issues as the primary reason for not using technology [14].

**Figure 1:**
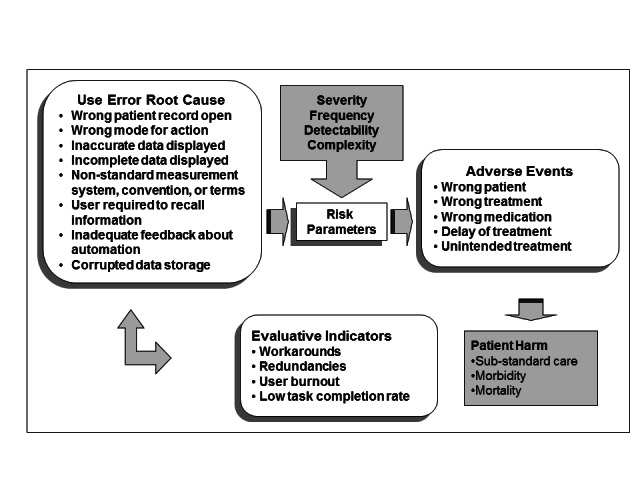
A model for analysis and understanding the use related risks of EHR systems.

Inappropriate embedded cultural assumptions that are associated with usability issues may also be associated with user and patient safety concerns as modeled by the EHR patient safety framework (Figure 1) [15-17]. This framework does not explicitly incorporate characteristics of EHR users such as culture, but it suggests that several types of design flaws induce use errors that can lead to patient harm. The frequency, detectability, and complexity of the user errors, as well as characteristics of patient populations, affect the potential magnitude of patient harm for a particular event [15-17]. Although this framework is designed to apply to EHR systems, we posit that it might also have utility with regard to EHR-associated patient portals and other consumer HIT.

Among HIT users who are members of racial and ethnic minority groups, it is possible that inappropriate embedded cultural assumptions in HIT may contribute to unique patient safety risks and/or concerns similar to risks stemming from off-label uses of medications that have not been tested with targeted patient populations. In other words, a design that is safe and effective for members of one population may create negative, unintended consequences for a population with different characteristics. A significant body of evidence from the medical, human factors, and ergonomics literature documents that these differing characteristics may be physical, cognitive, or cultural and that they can reduce the usability, usefulness, and safety of HIT systems [13,18,19].

## Methods

### Overview

Existing best practices, guidance, and standards in software usability and accessibility were subjected to expert review by the study team. An iterative consensus-based process was used to derive evidence-based design principles. The study team was composed of a physician researcher with expertise in health disparities and informatics and two human factors experts in informatics. The team developed and followed the 5-step methodology through regular monthly conference calls to discuss emerging findings and achieve consensus. Input from team members was delivered verbally and electronically in additional exchanges between meetings. Peer reviewers of a draft document included professionals with clinical expertise, informatics expertise, and human factors expertise. The purpose of this review was to identify those best practices, guidance, and standards that could help identify and/or address embedded design assumptions in HIT that could negatively impact patient safety, particularly for non-majority HIT user populations. In the Results section, we detail those principles derived from the human factors literature that underlie existing best practices, guidance, and standards in software usability and accessibility that could help identify and/or address embedded design assumptions in HIT that could negatively impact patient safety, particularly for HIT users who are members of non-majority groups. The 5-step methodology used to conduct the review and elicit applicable evidence is outlined below.

### Step 1

First, the target user populations were defined as potential EHR HIT user populations at increased baseline risk for health care disparities. As is clear from the health care literature, these persons include HIT users of low socioeconomic status, users who are members of racial and ethnic minority groups, users who are non-native English speakers or users with limited English proficiency, users with disabilities or who have physical or cognitive impairments, and persons older than 65 years of age.

### Step 2

Second, relevant risk characteristics of the target user populations that were likely to have EHR HIT design and/or usability correlates were identified. The culturally informed design framework describes 4 design dimensions (technology platform, functionality, content, and user interface) that are likely to influence HIT usability, acceptability, and effectiveness for a given cultural group. The culturally informed design framework also provides an evidence-based starting point for choosing those target population characteristics identified or hypothesized in the scientific literature to have EHR HIT design and/or usability correlates. The technology platform is defined as the type of HIT hardware in use. The functionality refers to the types of actions that may be performed within the HIT system. Content refers to the message delivered by the HIT system to the user. The user interface refers to the presentation and organization of the content and functionality associated with the hardware in question. Relevant risk characteristics of the target user populations were considered within these 4 domains.

### Step 3

#### Grounded Theory Approach

Third, a grounded theory approach was employed to identify potentially applicable best practices, guidance, and standards from industry and federal resources and from the scientific literature. The literature was reviewed and data extracted along the 4 design dimensions (technology platform, functionality, content, and user interface) suggested by the culturally informed design framework. The industry and federal resources and databases described below were assessed.

#### Standards of the International Organization for Standardization and International Electrotechnical Commission

The International Organization for Standardization (ISO) is the world’s largest developer of voluntary international standards. International standards give state-of-the-art specifications for products, services, and good practices to help make industry more efficient and effective. Developed through global consensus, they help to break down barriers to international trade. The ISO was founded in 1947 and since then has published more than 19,500 international standards covering almost all aspects of technology and business. In addition, the International Electrotechnical Commission (IEC) is a not-for-profit, non-governmental organization founded in 1906 that develops international standards and conducts conformity assessments for all electrical, electronics, and related technologies. Because many industries around the world, including the technology industry, rely on these organizations for guidance and standards, relevant reports from these organizations were reviewed as part of this study.

#### Section 508

In 1998, the US Congress amended the Rehabilitation Act of 1973 [20] to require federal agencies to make their electronic and information technology accessible to people with disabilities. Section 508 was enacted to eliminate barriers in IT, to make available new opportunities for people with disabilities, and to encourage development of technologies that help achieve these goals. The Access Board, created by the US Congress in 1973, is an independent federal agency devoted to accessibility for people with disabilities and is the federal agency responsible for development and dissemination of Section 508 technical standards. The board develops and maintains design criteria for the built environment, transit vehicles, telecommunications equipment, and electronic and information technology. It also provides technical assistance and training on these requirements and on accessible design, and it continues to enforce accessibility standards that cover federally funded facilities.

#### The World Wide Web Consortium

The World Wide Web Consortium (W3C) is an international community that develops open standards to ensure the long-term growth of the Web. Web content accessibility guidelines (WCAG) are developed through the W3C process in cooperation with individuals and organizations around the world with the goal of providing a single, shared standard for Web content accessibility that meets the needs of individuals, organizations, and governments internationally. The WCAG guidelines explain how to make Web content more accessible to people with disabilities.

#### The National Institute of Standards and Technology

Founded in 1901 and now part of the US Department of Commerce, the mission of the National Institute of Standards and Technology (NIST) is to promote US innovation and industrial competitiveness by advancing measurement science, standards, and technology in ways that enhance economic security and improve quality of life. One of the major research components of NIST is the Information Technology Laboratory (ITL), which has been charged with the task of utilizing existing and emerging IT to meet national priorities that reflect the country’s broad-based social, economic, and political values and goals. Its extended charge under the Federal Information Security Management Act [21] is to continue to develop cybersecurity standards, guidelines, and associated methods and techniques.

#### The Office of Minority Health

The Office of Minority Health (OMH) was created within the US Department of Health and Human Services in 1986. It is one of the most significant outcomes of the 1985 *Report of the Secretary's Task Force on Black and Minority Health* [22]. The OMH is dedicated to improving the health of racial and ethnic minority populations through the development of health policies and programs that will help eliminate health disparities. Among other things, the OMH develops and promotes policies, programs, and practices to eliminate health disparities and achieve health equity.

### Step 4

Fourth, all potential evidence extracted from the above-described literature was reviewed in detail. Inappropriate or otherwise inapplicable best practices, guidance, and standards were excluded on the basis of specific exclusion criteria. The exclusion criteria included (1) no plausible association between design feature guidance and a culturally based risk characteristic and (2) no available design enhancement to improve a design element that could contribute to HIT utilization disparities by increasing the risk of usability or patient safety challenges, particularly among the identified target user population.

### Step 5

Fifth, best practices, guidance, and standards guidance were summarized and underlying principles were derived. A consensus-based approach was employed in which each member of the study team could suggest a principle derived from the included literature. This principle was then discussed among study investigators. Principles were iteratively revised and amended. Final principles were included only if and when consensus was achieved. Consensus was sought regarding the state of the current evidence upon which the principle is based and the degree to which the principle provides actionable guidance.

## Results

### Overview

In reviewing the gathered documents, there appeared to be underlying principles from the human factors literature regarding how to discover unmet needs for a given population. In our review, the target population of interest included users of low socioeconomic status, users who are members of racial and ethnic minorities, users with limited English proficiency, persons older than 65 years of age, and users who have physical and/or cognitive impairments. It is important to realize that these principles do not apply exclusively to this target population. Rather, their importance is derived from the fact that, if significant attention is not given to these principles, the resulting HIT system designs may unintentionally result in avoidable usability and or patient safety challenges that differentially impact “non-typical” users.

### Principle 1.0: Information Technology Should Be Designed Based Upon a Model of Error and Expertise in Practice

In 1996, Woods, Patterson, and colleagues defined three levels of practice-centered design: (1) understanding, (2) usefulness, and (3) usability [23,24]. At the level of usability, the interface and information design is analyzed for how easy it is for representative users to accomplish designed tasks. At the level of usefulness, evaluations of prototypes generate new ideas for features and alternative approaches to meet newly discovered requirements. At the level of understanding, for practice-centered design, the problem definition is more than merely learning about the field of practice and talking to the practitioners; the designer must understand the nature of errors that occur and how experienced practitioners develop and maintain expertise. At the level of understanding, the typical discovery is that an embedded assumption in the design of an HIT system is not supported when representative users are asked to perform a task. For example, a user might be expected to forget a password needed to use an HIT system if it is complicated, but it might be discovered that a common strategy is to write passwords into a text message that is sent to themselves and always maintained on their telephone. Thus, a more likely issue would be for someone else to find the text message with the passwords to avoid forgetting the login information. Whether assumptions are due to culturally influenced expectations or other means impacts the predictability of anticipating issues, but the underlying principle of having a practice-centered design to support learning more about the nature of how errors are made and which strategies are employed to meet needs remains the same [25].

### Principle 2.0: Designing for Settings, Providers, and Users With Limited Resources Improves Usability for All Users

When designing HIT systems for persons with special needs or for those otherwise disadvantaged, a typical outcome is to reduce the resources required to easily use an HIT system. For example, rather than requiring two hands to use a device, a “swipe” method could be devised that would allow a task to be accomplished by using the same hand that holds a device. Similarly, a button could be enlarged to make it easier for an older adult user with tremor to tap, which also would make it easier for anyone to tap in a particular environment, such as while on a moving train. Therefore, in many cases, it is believed that designing HIT systems for populations with unique technological needs will not result in a design that is tailored in such a way that only a narrow user population would use it. In fact, it is expected that making interfaces easier to use for persons with lesser financial, cognitive, physical, and educational resources will result in the development of better HIT systems for all users. There will be exceptions for culturally informed design, such as culturally appropriate language and navigational expectations, but this is expected to form a small percentage of recommended changes when tailoring an HIT system to a target population.

### Principle 3.0: Authentication Is Often One of the Most Complex Elements of an HIT System

A standard heuristic in Web site design is to avoid or delay authentication steps because many users will not pass beyond that step. Similarly, a common measure of Web site usability is “completion rates,” which is defined based on the proportion of users who complete a task that has been started. Authentication as it is currently performed with HIT systems is often challenging, even for extremely experienced users with a high degree of savvy regarding new technologies. Workarounds that are likely to be found with authentication, such as using memory aids with written passwords, sharing passwords with family members, and using variants of a single password for multiple HIT systems, often exist in all populations. As resources become more limited, it is predictable that these workarounds will be found or even increased. HIT systems that require particularly complex or rigid password structures are unlikely to work well without workarounds for these populations, which may reduce the integrity of protecting the information. In addition, because the health and medical literature suggests that health and health care decision-making and behaviors occur within a broader social context, particularly among racial and ethnic minority group members [26-28], as well as in situations where there are complex and dynamic relationships with extended family members and caregivers and management of multiple levels of access. In such circumstances, the presence of multiple users accessing information will likely require enhanced support.

### Principle 4.0: Explicitly Design to Protect Against Undesired Use of Information by Unintended Users

For populations with a historical mistrust of the medical establishment, transparency about who is allowed to view private health information, as well as the protections provided for breaches of confidentiality, is critical. Without addressing this aspect of HIT, it is possible that useful HIT systems will fail to be adopted by particular populations. Although this level of mistrust may not be exhibited by majority populations, a general principle for any HIT system design is to identify 3 common categories of users: primary users, secondary users, and unintended users. Primary users are the target population that directly uses the interface. Secondary users generally access information in an aggregated fashion from a database of information generated by primary users, but typically do not enter or modify data without permission granted for individual users. Unintended users are users who could generate negative consequences by accessing the data. This does not necessarily involve illegal access to the data, such as by people trying to steal an identity. It could include legal discovery of information used in lawsuits or companies that use information for marketing purposes to identify trends that help them to place advertisements for targeted populations. Explicitly recognizing and mitigating the risks due to unintended (but predictable) uses of information is not a new idea in HIT system design. For example, features that generate reports from trends tracked on the basis of multiple users could be designed to automatically remove any information that might inadvertently identify a particular user who has sensitive information available in the database.

### Principle 5.0: Conduct Comprehensive Formative and Summative Testing With a Reasonable Set of Representative Target Users

With the burgeoning number of persons older than 65 years of age, rapidly growing numbers of immigrants with limited English proficiency, and surging numbers of racial and ethnic minorities projected for the US population over the next decade, the very notion of a “typical” user may need to be called into question. To help ensure broad accessibility and usability, it is imperative that testing involve both typical and non-typical users who are likely to use the HIT system. In addition to testing with representative users, it is important to use cases that allow the identification of culturally embedded assumptions that do not match the assumptions of intended users.

## Discussion

### Overview

To identify culturally embedded assumptions, we recommend that representative users from all anticipated markets for a product, including populations with unique technological needs, be sufficiently represented in summative usability evaluations and other design activities. Summative usability testing is generally conducted prior to implementation of an HIT system and involves having representative users interact with an HIT system to conduct tasks, including any tasks that are anticipated to be particularly challenging for any reason. Use cases are typically employed throughout the entire design cycle for any IT, from initial generation of rough mockups to summative usability evaluations. Explicitly designing use cases to support the discovery of embedded assumptions that would reduce willingness to adopt an HIT system or that could create a patient safety issue is recommended. Cultural differences in a majority population will be unique for a specific population of interest. Some variables to consider when characterizing a population with respect to HIT use include health literacy; IT literacy; socioeconomic status; level of influence on decision-making by health care providers, family members, and religious or community leaders; native language; English proficiency; prevalence of disabilities; age; race; ethnicity; home environment; geographic location; and country of origin.

We take low-income African Americans as one example of a group with special HIT needs. For this group, some differences from majority populations might include lower socioeconomic status, limited English proficiency, low health literacy, limited access to health care, and a high level of mistrust of the health care system [3]. To design HIT systems for this population, one recommendation is to include disparities-oriented use case scenarios and user contexts as part of the EHR HIT system design and developmental planning process. Examples of disparities-oriented use cases could include use cases that include one or more of the aspects described below.

### Safety Net Provider

Developing and applying use cases involving common tasks specific to clinical practice in safety net contexts are key to elucidating unrecognized user requirements. For example, a safety net provider may show a patient lab results by viewing them together on a desktop computer screen designed for the physician. In this use case, a patient may find it difficult to understand that hemoglobin A1c is a measure of blood sugar, for example. In addition, there are situations in which a patient with diabetes mellitus may be counseled to reduce blood sugar to a level below a normal range, and it may be difficult for physicians to explain the reason for this if the display labels a result as being within a normal range. An HIT system that does not address these embedded assumptions could lead to poor physician-patient communication and an increased likelihood of poor adherence to provider recommendations [13,29].

### Adult Caregiver of a Senior Relative

In many families, the need for adult children to care for elderly parents is becoming increasingly common. This may be even more likely among patients who are members of disadvantaged populations, who may lack resources to provide alternate care arrangements for their elderly relatives. The cognitive and physical demands and stress of caregiving, combined with childrearing, homemaking, and holding one or more jobs, may create critical challenges for the safe and effective use of EHRs. For example, consumer HIT systems may need to be designed to minimize disruption of elder care, such as by having an option to use it in a dark room without generating a bright light while an elderly parent is sleeping.

### Patients and Caregivers With Limited English Proficiency

Providing care to patients with limited English proficiency creates challenges for all involved parties. Employing a set of common “referent” terms and symbols that have been found to be common across cultures is recommended when available. When not available, including study participants within the range of a target population’s level of English proficiency during usability evaluations is recommended, as well as ensuring that complex terms and constructs are included in the use case scenarios.

### Electronic Health Record Use in the Context of Doctor–Patient Cross-Cultural or Communication Barriers

In some cultures, major decision-making is considered a family activity or at least a combined activity between spouses, family members, and sometimes close confidants (eg, clergy). However, informed consent and access to health information using a personal login is currently considered largely from a Western perspective (i.e., single users and individual rights). Usability, user experience, and user satisfaction implications with the application of such an EHR HIT system are not likely to be optimal. Design accommodations could help address the challenges created by these cultural differences, such as by allowing multiple users to employ the same login credentials to access information. In addition, representative user populations could be expanded to include all appropriate decision makers during the simulated sessions of a usability test, or study participants could be explicitly asked about which other members of a family or social network might play a role in decision-making and how they could best access information.

### Conclusions

We provide evidence-based examples and human factors–based principles to help HIT system designers recognize and address patient safety issues that may be due to inappropriate, embedded cultural assumptions in HIT. Matching embedded assumptions in designed HIT systems to cultural expectations of actual versus intended or perceived users will likely increase the usability, usefulness, and safety of HIT systems, particularly for user populations whose characteristics differ from those of the users anticipated by the original HIT system designers. In so doing, the likelihood of creating or exacerbating HIT system usability–related disparities would be significantly reduced. Much more work is needed, however, to empirically and definitively characterize the scope and magnitude of impact, as well as to rank the importance of usability issues, unique to populations whose characteristics differ from stereotypical users and to document the effectiveness of the design principles outlined in this review.
